# The Clinician as DIRECTOR: Operationalizing Cinematic Storytelling for Clinical Documentation in the Age of Artificial Intelligence

**DOI:** 10.7759/cureus.101523

**Published:** 2026-01-14

**Authors:** Shaheen E Lakhan

**Affiliations:** 1 Bioscience, Boricua Bio, San Juan, USA; 2 Bioscience, Global Neuroscience Initiative Foundation, Miami, USA; 3 Neurology, Western University of Health Sciences, Pomona, USA; 4 Adjunct Clinical Professor of Neurology, A.T. Still University School of Osteopathic Medicine in Arizona, Mesa, USA; 5 Adjunct Clinical Professor of Medicine, Morehouse School of Medicine, Atlanta, USA

**Keywords:** ambient ai, artificial intelligence, clinical documentation, clinical reasoning, electronic health records, medical education, narrative medicine, patient safety, storytelling

## Abstract

Clinical documentation is undergoing a structural transformation. Ambient artificial intelligence (AI) systems and large language models are increasingly capable of generating complete clinical notes from conversation transcripts, structured data, and templates, often with minimal clinician input. While these systems reduce clerical burden and improve efficiency, they introduce a new and underrecognized risk: erosion of narrative control. AI-generated notes frequently resemble unedited footage rather than finished stories, dense with information yet lacking framing, causality, prioritization, and closure. Film and television faced an analogous challenge decades ago, integrating fragmented scenes, multiple contributors, and extensive post-production editing into coherent narratives through formal storytelling mechanics. This article bridges cinematic storytelling principles and clinical documentation, reframing clinicians as directors rather than scribes in an AI-driven environment. We operationalize narrative constructs such as framing, stakes, conflict, continuity, resolution, and foreshadowing as practical tools for constructing and editing clinical notes. The DIRECTOR Framework defines the core narrative engine of documentation, while two Director-Level Overrides ensure ethical tone and authorship integrity. Multiple before-and-after examples of AI-generated notes demonstrate how minimal clinician intervention can restore reasoning, preserve human intent, and improve patient safety. In the era of ambient AI, storytelling is not a metaphor for documentation. It is the control layer that keeps the medical record intelligible, defensible, and humane.

## Editorial

Introduction

Clinical documentation has always served multiple purposes, but never to the degree seen today. The modern clinical note is expected to support patient care, billing, quality reporting, legal defensibility, patient transparency, and data extraction, often simultaneously. Electronic health records enabled this expansion by making data capture easier and more comprehensive. Ambient artificial intelligence (AI) now accelerates it further by automating note generation itself [1].

As a result, documentation has shifted from authored narrative to assembled artifact. Notes are increasingly generated by systems that listen continuously, extract structured elements, and synthesize fluent text. In many settings, clinicians are no longer writing notes so much as reviewing them [2].

This shift is often framed as progress. Yet it fundamentally alters the nature of the medical record. Historically, the clinical note was a cognitive artifact, a written expression of clinical reasoning. It explained why certain diagnoses were considered, why others were rejected, and why a particular plan was chosen at a particular moment. That explanatory function is now at risk.

AI-generated notes are frequently accurate in content yet ambiguous in meaning [3]. They document what was said without reliably capturing what mattered. They prioritize coherence over uncertainty and fluency over intent. The danger is not that they are wrong, but that they are convincingly incomplete.

Film and television confronted this problem long before medicine did. Large productions generate enormous volumes of raw material. Scenes are filmed out of order, edited repeatedly, and shaped by many contributors. Without narrative discipline, the result would be incoherence. Cinema solved this problem by formalizing storytelling mechanics and assigning responsibility for narrative coherence to a single role: the director.

This paper argues that clinical documentation now requires the same discipline. In an AI-driven environment, clinicians must function as directors of meaning rather than scribes of text. Cinematic storytelling principles provide a practical, scalable framework for restoring coherence, intent, and safety to the medical record.

Why cinema is the correct analogy for AI-Driven documentation

The analogy between cinema and clinical documentation is structural rather than metaphorical. Both domains involve complex production pipelines, multiple stakeholders, and post-production editing that can radically alter meaning.

In modern film production, raw footage is abundant and inexpensive. Meaning is created through selection, sequencing, emphasis, and resolution. Editors and algorithms can assemble scenes, but they cannot determine narrative priority. That responsibility remains human.

Clinical documentation now operates similarly. Ambient AI systems generate transcripts. EHRs assemble data. Templates impose structure. What is missing is narrative governance. Without it, notes become compilations rather than arguments.

Cinema teaches that coherence does not emerge automatically from completeness. In fact, completeness without structure often obscures meaning. This insight applies directly to clinical notes that contain every data point but fail to explain decisions.

The clinician as director rather than scribe

Historically, clinicians functioned as both camera operator and director. They observed, interpreted, and documented. Automation has separated these roles. AI systems now capture and assemble content, functioning as camera crews and editors.

The clinician’s role must therefore evolve. The clinician is no longer responsible for typing every word. The clinician is responsible for ensuring that the final product reflects intent, reasoning, and continuity.

In cinema, directors do not edit every frame. They review cuts, identify narrative gaps, and demand revisions. They ensure that the final version tells the right story. Clinical documentation now requires the same mindset.

This reframing clarifies accountability. If an AI-generated note misrepresents clinical reasoning, responsibility still rests with the clinician who approved it. Acting as director acknowledges that reality and provides a framework for fulfilling it.

AI as post-production, not author

AI excels at post-production tasks. It can summarize, reorder, harmonize, and polish. These are editorial functions. What AI cannot reliably do is determine narrative importance [4].

Left unsupervised, AI introduces predictable distortions. It resolves ambiguity prematurely because uncertainty degrades coherence. It smooths contradictions rather than surfacing them. It may infer causality where none was intended.

These distortions are analogous to aggressive editing that removes narrative tension from a film. The result may be fluent, but it is dishonest.

Recognizing AI as post-production rather than authorship clarifies its appropriate role. The clinician supplies narrative anchors. AI renders them legible.

Core cinematic storytelling principles and their clinical equivalents

Cinematic storytelling relies on a small set of universal mechanics. Each has a direct analogue in clinical documentation.

Framing establishes what the scene is about. In clinical notes, framing answers why this encounter mattered now. Stakes define why the story matters and correspond to clinical risk. Conflict drives the narrative forward and maps directly to the differential diagnosis. Causality connects observations to decisions. Resolution closes narrative loops. Foreshadowing anticipates future developments and contingency planning.

These elements are not stylistic choices. They are the minimum structure required for meaning.

The DIRECTOR framework for clinical documentation

These principles are operationalized as the DIRECTOR Framework, which defines the core narrative engine of clinical documentation (Table 1). DIRECTOR stands for the following: D - Define the encounter; I - Identify the stakes; R - Resolve the differential; E - Emphasize signal over noise; C - Connect findings to decisions; T - Track continuity over time; O - Own unresolved threads; R - Record contingencies.

**Table 1:** The DIRECTOR Framework and Director-Level Overrides for Clinical Documentation in the Age of AI The DIRECTOR Framework defines the core narrative structure of clinical documentation. Director-Level Overrides ensure ethical tone and preservation of clinician intent in AI-generated notes.

Level	Narrative Element	Guiding Question	Practical Action
DIRECTOR	Define the Encounter	What is this scene really about	State the decision this visit addressed
	Identify the Stakes	What happens if this goes wrong	Document the most serious outcome considered
	Resolve the Differential	What are the competing storylines	Name a serious alternative and why it was deprioritized
	Emphasize Signal	Which details advance the plot	Clarify which findings influenced reasoning
	Connect Decisions	Why does one event lead to another	Link findings directly to actions
	Track Continuity	Does this match prior scenes	Correct copied-forward elements
	Own Unresolved Threads	Are story threads closed	Close or explicitly defer findings
	Record Contingencies	What could change the ending	Document reassessment triggers
Override	Tone Control	Is the narrator trustworthy	Remove stigmatizing or opaque language
	Director’s Cut	Does this reflect my intent	Confirm clinician authorship of judgment

In practice, applying the DIRECTOR Framework requires less than 30 seconds. Clinicians do not rewrite notes. They insert one framing sentence, one risk acknowledgment, and one contingency statement. Most AI-generated notes already contain the necessary raw material. The framework functions as a final narrative pass rather than a documentation burden.

Importantly, this approach replaces redundant behaviors such as excessive copy-forward, exhaustive data repetition, and defensive verbosity [5].

Director-Level Overrides

The DIRECTOR Framework defines the narrative engine, but two additional checks function at a supervisory level. These Director-Level Overrides ensure ethical tone and authorship integrity and are applied after the core framework.

Tone Control ensures that patient-facing language preserves trust, avoids stigmatization, and accurately reflects uncertainty.

Director’s Cut is the final authorship check, confirming that the note reflects clinician judgment rather than AI inference.

These overrides mirror cinematic practice, where final edits ensure tone consistency and directorial intent before release.

Before-and-after examples: directing AI-generated notes in practice

The following examples illustrate how cinematic storytelling principles and the DIRECTOR Framework can be applied in real clinical settings. In each case, the initial note represents a plausible AI-generated assessment that is fluent, complete, and technically accurate, yet narratively unsafe. The revised version demonstrates how minimal clinician edits, often limited to two or three sentences, restore framing, reasoning, and continuity.

Example 1: Emergency Department Evaluation for Chest Pain

AI-generated assessment (before): The patient presents with chest pain and shortness of breath. Vital signs are stable. ECG is normal. Troponin is negative. CT angiography is negative. Symptoms may be related to anxiety. Plan is discharge with reassurance and outpatient follow-up.

This note resembles unedited footage. It lists findings and conclusions but provides no narrative structure. The primary clinical question is unstated, the stakes are invisible, and the rationale for excluding a potentially life-threatening diagnosis is implicit rather than explicit. To a future reader, the absence of concern may appear intentional rather than unarticulated.

Clinician-directed assessment (after): The primary concern for this encounter was exclusion of pulmonary embolism in the setting of acute chest pain and dyspnea. Despite stable vital signs and normal ECG and troponin, pulmonary embolism was considered due to the acuity of symptoms. CT angiography was negative, making this diagnosis unlikely at this time. Symptoms are therefore felt to be non-cardiopulmonary in origin. The patient was discharged with strict return precautions, including worsening chest pain or dyspnea, which would prompt immediate reevaluation.

In this version, the clinician directs the narrative by defining the encounter, identifying the stakes, resolving the differential, and recording contingencies. No new data are introduced. The note now explains itself. If the patient returns or experiences an adverse outcome, the record demonstrates deliberate reasoning rather than omission.

Example 2: Outpatient Follow-Up for Chronic Disease Management

AI-generated assessment (before): The patient is here for follow-up of diabetes and hypertension. Labs show HbA1c of 8.2 percent. Blood pressure today is 138 over 86. Medications reviewed. Continue current regimen.

This note is concise and factually correct but narratively inert. It documents measurements without interpretation and decisions without rationale. The reader cannot determine whether inaction reflects clinical judgment or oversight.

Clinician-directed assessment (after): This visit focused on reassessing glycemic control in a patient with type 2 diabetes whose HbA1c has increased to 8.2 percent. Although blood pressure remains near goal, worsening glycemic control raises concern for increased long-term cardiovascular risk. Medication escalation was deferred due to recent adherence challenges, with a plan to reassess after targeted lifestyle intervention. If HbA1c remains above target at the next visit, pharmacologic intensification will be initiated.

Here, the clinician defines the encounter, articulates the stakes, connects findings to decisions, and foreshadows next steps. The decision to defer treatment escalation is no longer ambiguous. It is intentional, bounded, and auditable.

Example 3: Inpatient Progress Note With Copy-Forward Risk

AI-generated assessment (before): The patient is resting comfortably. No acute events overnight. Continue current management.

In isolation, this sentence appears benign. In context, it may contradict vital sign trends, nursing notes, or evolving clinical instability. AI systems often perpetuate this language through copy-forward behavior, creating dangerous continuity errors [5].

Clinician-directed assessment (after): Although the patient reports improved comfort, overnight vitals demonstrated intermittent hypoxia requiring escalation to high-flow oxygen. Respiratory status remains the primary concern today. If oxygen requirements increase further or mental status worsens, transfer to a higher level of care will be pursued.

This edit restores continuity and signal prioritization. It reconciles conflicting inputs and records escalation criteria. The narrative now reflects the clinician’s situational awareness rather than the system’s default phrasing.

Summary of narrative intervention

Across these examples, the clinician does not rewrite the note or expand it substantially. Instead, the clinician performs a directorial function, shaping AI-generated content into a coherent story by explicitly stating intent, risk, reasoning, and contingency. These interventions require seconds, not minutes, yet fundamentally change the safety and interpretability of the medical record.

In an AI-dominated documentation environment, these narrative corrections are not optional refinements. They are the mechanisms by which clinical judgment remains visible.

Humanism through structure rather than sentiment

Humanism in documentation does not require emotive language. It requires transparency about uncertainty, concern, and judgment. AI-generated notes often sound reassuring while concealing complexity.

Patients are not harmed by clarity. They are harmed by silence.

Educational and health system implications

Narrative competence is diagnostic competence. Training programs should explicitly teach narrative governance using the DIRECTOR Framework. Health systems can operationalize this approach by requiring clinician-authored narrative anchors before note finalization.

Narrative quality can be assessed by the presence of framing, documented risk consideration, and contingency planning. These elements are measurable and auditable.

Conclusion

As AI increasingly generates the medical record, the central risk is no longer clerical error but narrative abdication. When clinicians approve AI-generated notes without explicitly directing meaning, they unintentionally delegate judgment itself. Film and television demonstrate that narrative coherence can survive industrial-scale automation when responsibility for the story remains clearly human. Clinical documentation now requires the same discipline. The clinician’s role is not to compete with AI in transcription or fluency, but to govern relevance, priority, and causality.

Storytelling in medicine is not an aesthetic preference or a nostalgic holdover from pre-digital practice. It is the mechanism by which reasoning becomes visible, risk becomes legible, and accountability is preserved. In the age of AI, the clinical note must explain not only what happened, but why it mattered and what could happen next. That responsibility cannot be automated, and it cannot be delegated.
